# Effects of Developmental Activation of the AhR on CD4^+^ T-Cell Responses to Influenza Virus Infection in Adult Mice

**DOI:** 10.1289/ehp.1408110

**Published:** 2014-07-22

**Authors:** Lisbeth A. Boule, Bethany Winans, B. Paige Lawrence

**Affiliations:** 1 Department of Microbiology and Immunology, and; 2 Department of Environmental Medicine, University of Rochester, Rochester, New York, USA

## Abstract

Background: Epidemiological and animal studies indicate that maternal exposure to pollutants that bind the aryl hydrocarbon receptor (AhR) correlates with poorer ability to combat respiratory infection and lower antibody levels in the offspring. These observations point to an impact on CD4^+^ T cells. Yet, the consequence of developmental exposure to AhR ligands on the activation and differentiation of CD4^+^ T cells has not been directly examined.

Objectives: Our goal was to determine whether maternal exposure to an AhR ligand directly alters CD4^+^ T cell differentiation and function later in life.

Methods: C57BL/6 mice were exposed to a prototypical AhR ligand, 2,3,7,8-tetrachlorodibenzo-*p*-dioxin (TCDD), *in utero* and via suckling. We then measured CD4^+^ T-cell activation and differentiation into distinct effector populations in adult offspring that were infected with influenza A virus (IAV). Reciprocal adoptive transfers were used to define whether modifications in CD4^+^ T-cell responses resulted from direct effects of developmental TCDD exposure on CD4^+^ T cells.

Results: Developmental exposure skewed CD4^+^ T-cell responses to IAV infection. We observed fewer virus-specific, activated CD4^+^ T cells and a reduced frequency of conventional CD4^+^ effector-cell subsets. However, there was an increase in regulatory CD4^+^ T cells. Direct effects of AhR activation on CD4^+^ T cells resulted in impaired differentiation into conventional effector subsets; this defect was transferred to mice that had not been developmentally exposed to TCDD.

Conclusions: Maternal exposure to TCDD resulted in durable changes in the responsive capacity and differentiation of CD4^+^ T cells in adult C57BL/6 mice.

Citation: Boule LA, Winans B, Lawrence BP. 2014. Effects of developmental activation of the AhR on CD4^+^ T-cell responses to influenza virus infection in adult mice. Environ Health Perspect 122:1201–1208; http://dx.doi.org/10.1289/ehp.1408110

## Introduction

Prenatal and early-life environmental factors, including exposure to exogenous chemicals, have been linked to increased risk of cancer, diabetes, cardiovascular disease, and obesity (Boekelheide et al. 2012). Although the immune system has been the focus of fewer studies, maternal exposures have been reported to influence immune responses (Winans et al. 2011). The consequences of alterations to the immune system are potentially serious because even subtle changes can diminish resistance to infections and reduce responses to vaccines. In fact, several recent reports suggest that these are real-world consequences of developmental exposures. For example, maternal and cord blood levels of polychlorinated biphenyls and dioxins correlate with decreased responses to routine vaccinations (Heilmann et al. 2010) and increased respiratory infections in children (Dallaire et al. 2006; Glynn et al. 2008; Hochstenbach et al. 2012; Stølevik et al. 2013). Exposure to these chemicals occurs regularly through the diet, and it has been estimated that fetuses and infants are exposed to higher levels due to bioaccumulation (Institute of Medicine 2003; Schecter et al. 2001). However, the cellular targets and mechanisms by which developmental exposures cause persistent changes in the function of the immune system are unknown.

CD4^+^ T cells are critical immune effector cells, and alteration in their function can have grave consequences on responses to primary infection and the acquisition of immunity. Infection initiates naive CD4^+^ T cells to differentiate into phenotypically and functionally distinct subsets, although the precise subset depends on particular pathogen-derived and tissue-specific cues (Yamane and Paul 2013). T helper 1 (Th1) and T follicular helper (Tfh) cells are two major conventional CD4^+^ effector subsets elicited by respiratory infection (Boyden et al. 2012; Chapman et al. 2005). Th1 cells produce the cytokine interferon gamma (IFNγ), and Tfh are critical for T-cell–dependent B-cell responses. Although their precise role during infection is not fully understood, Th17 cells correlate with reduced mortality in mice and humans (Almansa et al. 2011; McKinstry et al. 2009). Th2 cells contribute to responses to parasites and many allergic diseases, but they represent a small fraction of CD4^+^ effectors during respiratory viral infections. Th1, Tfh, Th17, and Th2 cells are considered conventional CD4^+^ T cells, whereas regulatory CD4^+^ T cells (Tregs) maintain peripheral tolerance and down-regulate responses in the context of numerous infections (Fontenot and Rudensky 2005). Changing the capacity of CD4^+^ T cells to differentiate into distinct effector subsets has major implications on the progression and resolution of infection.

Exposure to aryl hydrocarbon receptor (AhR) ligands alters CD4^+^ T-cell differentiation and function in developmentally mature organisms. For example, AhR ligands modulate conventional CD4^+^ T-cell responses, altering the proportion of Th1, Th2, and Th17 cells (Quintana and Sherr 2013). Direct treatment with AhR ligands also alters the frequency of Tregs, but often in the opposite direction from that of conventional CD4^+^ T cells, resulting in a greater frequency of Tregs (Quintana and Sherr 2013). Collectively, these studies indicate that exposure of the fully mature immune system to AhR ligands changes the proportion of functionally distinct effector subpopulations of CD4^+^ T cells and influences disease outcome. In contrast, the consequences of AhR activation during development on CD4^+^ T cells later in life have not been empirically studied. Yet, several pieces of evidence suggest that developmental exposure affects CD4^+^ T cells. First, developmental exposure to AhR ligands has been reported to decrease antibody responses to sheep erythrocytes and influenza A virus (IAV) (Thomas and Hinsdill 1979; Vorderstrasse et al. 2006) and reduce delayed-type hypersensitivity responses in adult offspring (Gehrs and Smialowicz 1999). Developmental exposure to 2,3,7,8-tetrachlorodibenzo-*p*-dioxin (TCDD) increased mortality after infection with *Listeria monocytogenes* (Sugita-Konishi et al. 2003) and enhanced susceptibility to tumor challenge (Luster et al. 1980). These processes all depend on the function of CD4^+^ T cells; however, whether CD4^+^ T-cell function is actually altered by AhR activation during development has not been directly examined.

In the present study, we examined whether AhR activation during development changes the response of CD4^+^ T cells to infection with IAV later in life. Specifically, following maternal exposure to an oral dose of TCDD that reportedly does not cause thymic or bone marrow hypocellularity or other signs of toxicity (Vorderstrasse et al. 2004), we determined the frequency of conventional, activated, and virus-specific CD4^+^ T cells. Further, we examined regulatory CD4^+^ T cells and the ratio of conventional:regulatory CD4^+^ T cells. Then, using reciprocal adoptive transfers, we evaluated whether CD4^+^ T-cell responses to infection were modulated via intrinsic or extrinsic effects of AhR activation during development on CD4^+^ T cells in adult mice. Delineating which aspects of CD4^+^ T-cell function were intrinsically altered by developmental exposure furthers our understanding of how AhR ligands may cause durable changes to CD4^+^ T-cell responses, and expands current understanding of how developmental exposures to AhR ligands may affect the immune responses later in life.

## Materials and Methods

*Animal treatment and cell preparation*. We obtained C57BL/6 (B6, CD90.2^+^CD45.2^+^), B6-LY5.2/Cr (CD90.2^+^CD45.1^+^), and B6.PL-*Thy1^a^*/CyJ (CD90.1^+^CD45.2^+^) mice (age 5 weeks) from the NCI (National Cancer Institute) Mouse Repository (Frederick, MD) or the Jackson Laboratory (Bar Harbor, ME); all mice were *Ahr*^+/+^. A colony of B6.AhR^tm1Bra^ (*Ahr*^–/–^) mice maintained at the University of Rochester Medical Center (URMC) has been described previously (Teske et al. 2005). After spending at least 1 week in the vivarium at the URMC, nulliparous females (8–10 weeks of age) were housed with males and checked daily for the presence of a vaginal plug (day 0 of gestation). All offspring (regardless of experimental end point measured) remained with their mothers until weaning at 20–21 days of age and were then housed in with same-sex littermates. Mice were housed in washed polysulfone microisolator cages in a specific-pathogen free facility, with controlled light (12 hr light/dark), temperature, and humidity, and were provided standard mouse chow (Autoclavable Rodent Diet 5010; LabDiet, St. Louis, MO) and water *ad libitum*. We used C57BL/6 (B6, CD90.2^+^CD45.2^+^) mice for all time-course studies and examination of CD4^+^ T-cell subsets; *Ahr*^–/–^ (B6.AhR^tm1Bra^) mice crossed with C57BL/6 mice (*Ahr*^+/+^) for experiments to determine whether offspring need to express AhR in order to experience changes due to TCDD exposure during development; and C57BL/6 (B6, CD90.2^+^CD45.2^+^), B6-LY5.2/Cr (CD90.2^+^CD45.1^+^), and B6.PL-*Thy1^a^*/CyJ (CD90.1^+^CD45.2^+^) mice for adoptive transfer studies.

Impregnated female mice were treated with 1 μg/kg body weight of TCDD (≥ 99% purity; Cambridge Isotope Laboratories, Woburn, MA) or peanut oil (vehicle) by gavage in the afternoon on days 0, 7, and 14 of gestation and again at 2 days after parturition (Vorderstrasse et al. 2004). The concentration of TCDD stock was maintained at 1 μg/mL in peanut oil so that dosing required 10 μL TCDD solution (or peanut oil vehicle) per gram of body weight. The time to parturition, litter size, and sex distribution of the offspring were not changed by exposure to this dose of TCDD (data not shown; Vorderstrasse et al. 2004).

IAV strain HK×31 (H3N2) was prepared, titered, and stored at –80°C as previously described (Warren et al. 2000). At 6–8 weeks of age, adult offspring that had been developmentally exposed to TCDD or peanut oil vehicle were anesthetized by intraperitoneal injection of avertin (2,2,2-tribromoethanol; Sigma Aldrich, Milwaukee, WI) and infected intranasally with 120 hemagglutinating units of IAV (Warren et al. 2000). For the time-course experiments, all mice were infected on the same day, and groups of mice were sacrificed at each time point. Naive (day 0 postinfection with IAV) adult offspring were included as controls in experiments quantifying the number of CD4^+^ T cells and examining the IAV-specific antibody response. In the morning of each day postinfection (e.g., day 3, 6, 9 or 12 postinfection, spleens and lymph nodes were removed, and single cell suspensions were made. Blood was collected into heparin-loaded syringes via cardiac puncture, and erythrocytes were removed by hypotonic lysis. Unless otherwise specified, four to six female offspring from separate dams were used at each time point for each treatment group. All animal treatments and work with infectious agents were conducted with prior approval of the Institutional Animal Care and Use Committee and the Institutional Biosafety Committee of the University of Rochester. All animals used were treated humanely and with regard for alleviation of suffering.

*Flow cytometry*. To define specific populations of cells, including CD4^+^ T cells, effector CD4^+^ T cells (CD44^hi^CD62L^lo^), Tfh cells (CD4^+^CD44^hi^CXCR5^+^PD-1^+^), germinal center B cells (GL-7^+^CD95^+^B220^+^), and plasma cells (B220^int^CD138^+^), isolated cells were co-labeled with fluorochrome-conjugated antibodies to specific cell-surface molecules (Jin et al. 2014; Vorderstrasse et al. 2004; Wheeler et al. 2013). Cells were incubated with major histocompatibility class II tetramers containing an immunodominant peptide epitope of HK×31 (nucleoprotein, I-A^b^/NP_311–325_; NIH Tetramer Core Facility, http://tetramer.yerkes.emory.edu/). For intracellular molecules, cells were fixed, permeabilized, and co-incubated with antibodies against Foxp3 (forkhead box protein P3), GATA3 (GATA binding protein 3), RORγt (retinoid-related orphan receptor gamma t), and Tbet (T-box transcription factor TBX21). Nonspecific staining was blocked using anti-mouse CD16/32 monoclonal antibody. All antibodies were obtained from BD Biosciences (San Diego, CA) or eBiosciences (San Diego, CA). Data were collected using an LSRII flow cytometer (BD Biosciences), and analyzed using FlowJo software (TreeStar, Ashland, OR). Fluorescence minus one (FMO) was used to define gating parameters. Details regarding gating strategies and cell number calculations are provided in Supplemental Material, Figure S1. We used data from individual animals in all experiments; however, for adoptive transfer experiments, data were concatenated due to the low number of gated events for T-cell subsets. Concatenation was performed after analysis to better visualize our findings; it did not affect the distribution of the data or conclusions.

*Enzyme-linked immunosorbent assay (ELISA) and enzyme-linked immunosorbent spot (ELISPOT) assays.* IAV-specific antibodies (IgG2a and IgM) were detected in serum collected on day 9 after infection, using an isotype-specific ELISA (Vorderstrasse et al. 2006). IFNγ-producing CD4^+^ T cells were enumerated by ELISPOT assay. Briefly, CD4^+^ T cells were negatively enriched with a MagCellect Mouse CD4^+^ T-cell isolation kit (R&D, Minneapolis, MN), serially diluted starting with 1 × 10^5^ cells/well, and added to 96-well plates (Millipore, Bedford, MA) that had been coated with anti-IFNγ antibody (Mabtech, Nacka, Sweden). Virus-pulsed, irradiated DC2.4 cells (provided by K. Rock, Dana Farber Cancer Institute, Boston, MA) were used as antigen-presenting cells (5 × 10^4^ cells/well). Biotinylated anti-IFNγ antibody (Mabtech) and avidin–alkaline phosphatase (Southern Biotech, Birmingham, AL) were added, and spots were visualized using the Vector Blue Alkaline Phosphatase Substrate Kit (Vector Laboratories, Burlingame, CA). Spots were counted using a CTL plate reader and Immunospot software (Cellular Technologies, Shaker Heights, OH).

*Adoptive transfers*. CD4^+^ T cells collected from peripheral lymph nodes of mice were negatively enriched and sorted (FACSAria, BD Biosciences) to obtain CD44^lo^ (naive) CD4^+^ cells (≥ 95% purity). CD44^lo^CD4^+^ cells (5 × 10^5^) from 5–7 mice were diluted in phosphate-buffered saline and transferred intravenously (200 μL) into recipient mice (5–10 recipients per experiment). For dual adoptive transfers, CD44^lo^CD4^+^ cells from offspring of TCDD- or vehicle-treated dams were combined and co-transferred (i.e., recipients received 2.5 × 10^5^ cells from each donor; 200 μL total volume). For the single adoptive transfer, naive CD4^+^ T cells (5 × 10^5^ in a total volume of 200 μL) from unexposed donors were transferred into congenic adult offspring of dams that were treated with either vehicle or TCDD. For both adoptive transfer systems, at 36 hr posttransfer, cells from blood, peripheral lymph nodes, and spleen were harvested from a subset of recipients, and the remainder of recipients were infected with IAV. Cells from the IAV-infected recipients were harvested 9 days after infection.

*Statistical analysis*. We used the dam as the statistical unit for all experiments; thus, offspring in each treatment group and at each time point relative to infection were from a different treated dam. Data were analyzed using JMP software (SAS Institute Inc., Cary, NC). Differences between two groups at a single time point were evaluated using Student’s *t*-test. We used a one-way or two-way analysis of variance, followed by a Tukey multiple comparisons *post hoc* test, to compare differences between offspring of treated dams over time or at various dilutions of cells or serum. Differences were considered significant at *p* < 0.05, and all data are presented as mean ± SE. All experiments were independently repeated at least one time, with similar results.

## Results

**Figure 1:**
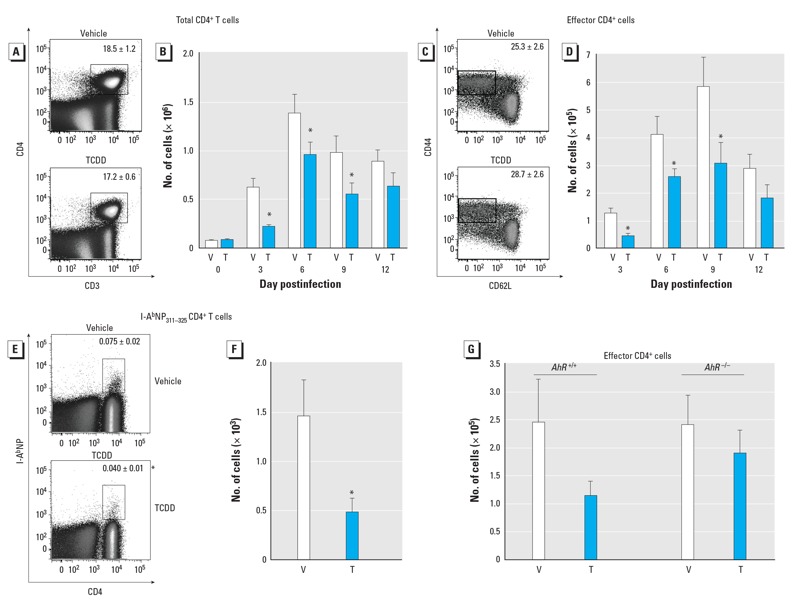
Developmental activation of the AhR diminished the virus-specific CD4^+^ T‑cell response to infection. MLN cells from adult mice developmentally exposed to vehicle (V) or TCDD (T) and infected intranasally with IAV were stained for flow cytometry. The percentage (*A*) and number (*B*) of CD4^+^ T cells over time, and the percentage (*C*) and number (*D*) of effector (CD44^hi^CD62L^lo^) CD4^+^ T cells over time. Representative plots (*A*, *C*) are from day 3 and day 9 postinfection, respectively. The percentage (*E*) and number (*F*) of I-A^b^NP_311–325_^+^CD4^+^ T cells on day 9 postinfection. (*G*) The number of CD44^hi^CD62L^lo^CD4^+^ T cells in *AhR^+/+^* and *AhR^–/–^* offspring. Values shown are mean ± SE; five or six female offspring from separate dams were used per group at each time point. *p ≤ 0.05.

*CD4^+^ T-cell responses in developmentally exposed offspring*. Respiratory infections trigger clonal expansion and differentiation of pathogen-specific T lymphocytes, a process that largely takes place in the secondary lymphoid organs that drain the respiratory tract, such as the mediastinal lymph nodes (MLN). Prior to infection, the number of CD4^+^ T cells in the MLN was not different in naive mice (day 0 postinfection) developmentally exposed to vehicle or TCDD (Figure 1A). However, after infection there was a significant reduction in the number, but not the percentage, of CD4^+^ T cells in MLN of mice developmentally exposed to TCDD (Figure 1A,B). This reduction persisted until 9 days after infection. We also observed a decrease in number—but not percentage—of CD44^hi^CD62L^lo^CD4^+^ cells, indicating fewer activated effector CD4^+^ T cells (Figure 1C,D). This decrease in effector CD4^+^ T cells was due to the reduction in the total number of CD4^+^ T cells (Figure 1B). In addition, the frequency of CD4^+^ T cells specific for a dominant IAV epitope (a viral nucleoprotein-derived peptide, NP_311–325_) was reduced by 50% in infected offspring of TCDD-treated dams (Figure 1E,F). Using *AhR^+/–^* dams, we confirmed that the presence of the AhR was required in the offspring in order for maternal treatment with TCDD to alter the CD4^+^ T-cell response to infection (Figure 1G). Only infected *AhR^+/+^* offspring from TCDD-treated dams had a reduced number of effector CD4^+^ T cells, whereas *AhR^–/–^* littermates from TCDD-treated dams did not. Male and female adult offspring showed no differences in their responses to IAV infection, and both males and females exhibited the same changes after TCDD exposure during development (Vorderstrasse et al. 2004).

**Figure 2:**
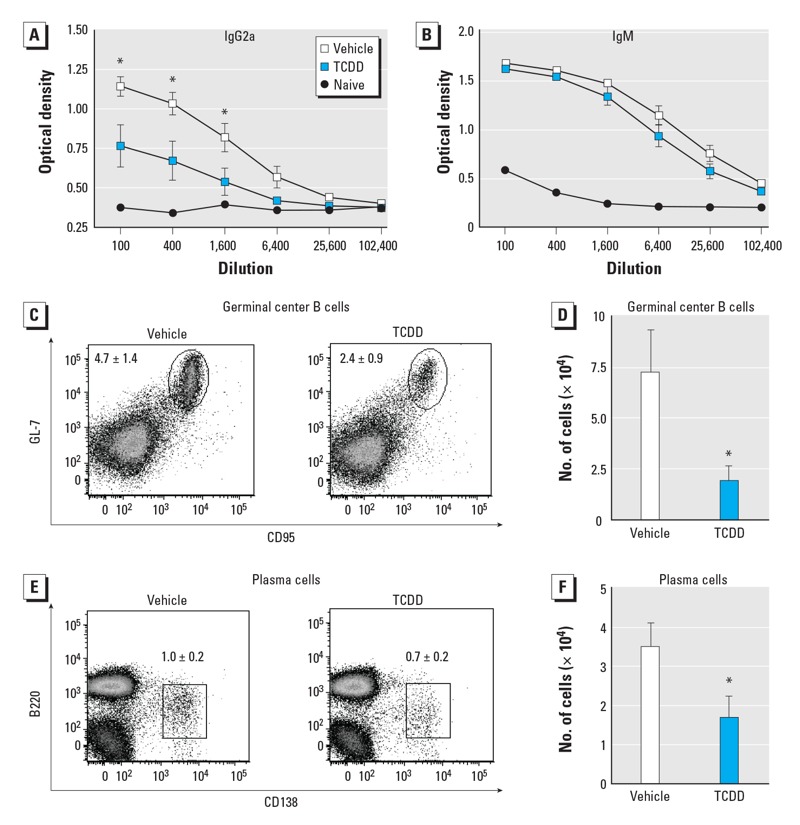
The virus-specific, class-switched antibody response to IAV was reduced by activation of the AhR during development. Mice were developmentally exposed to vehicle (V) or TCDD (T) and infected with IAV as adults. IAV-specific IgG2a (*A*) and IgM (*B*) levels in serially diluted serum determined by ELISA on day 9 after infection; serum from unexposed, naive mice served as the control. The percentage (*C,E*) and number (*D,F*) of germinal center B cells (B220^+^CD95^+^GL-7^+^; *C,D*) and plasma cells (CD138^+^B220^int^; *E,F*) in the MLN on day 9 postinfection quantified by flow cytometry. Values shown are mean ± SE; four or five female mice from separate dams were used for each group. *p ≤ 0.05.

An important role of CD4^+^ T cells during infection is aiding in the formation of a robust virus-specific antibody response (Alam et al. 2014). Thus, we compared the effects of developmental TCDD exposure on CD4^+^ T-cell–dependent and independent influenza-specific antibody levels, and on CD4^+^ T-cell–dependent B-cell differentiation. Consistent with prior reports (Vorderstrasse et al. 2006), offspring of dams exposed to TCDD had significantly reduced virus-specific IgG2a levels compared with offspring of vehicle-treated dams (CD4^+^ T-cell dependent; Figure 2A). In contrast, levels of virus-specific IgM (CD4^+^ T-cell independent) were not different (Figure 2B). To further examine CD4^+^ T-cell–dependent B-cell responses, we determined the frequency of germinal center B cells and plasma cells in MLN. The overall frequency of germinal center B cells (Figure 2C,D) and plasma cells (Figure 2E,F) was reduced by 2- to 3-fold in infected adult offspring of TCDD-treated dams compared with those of control-treated dams.

**Figure 3:**
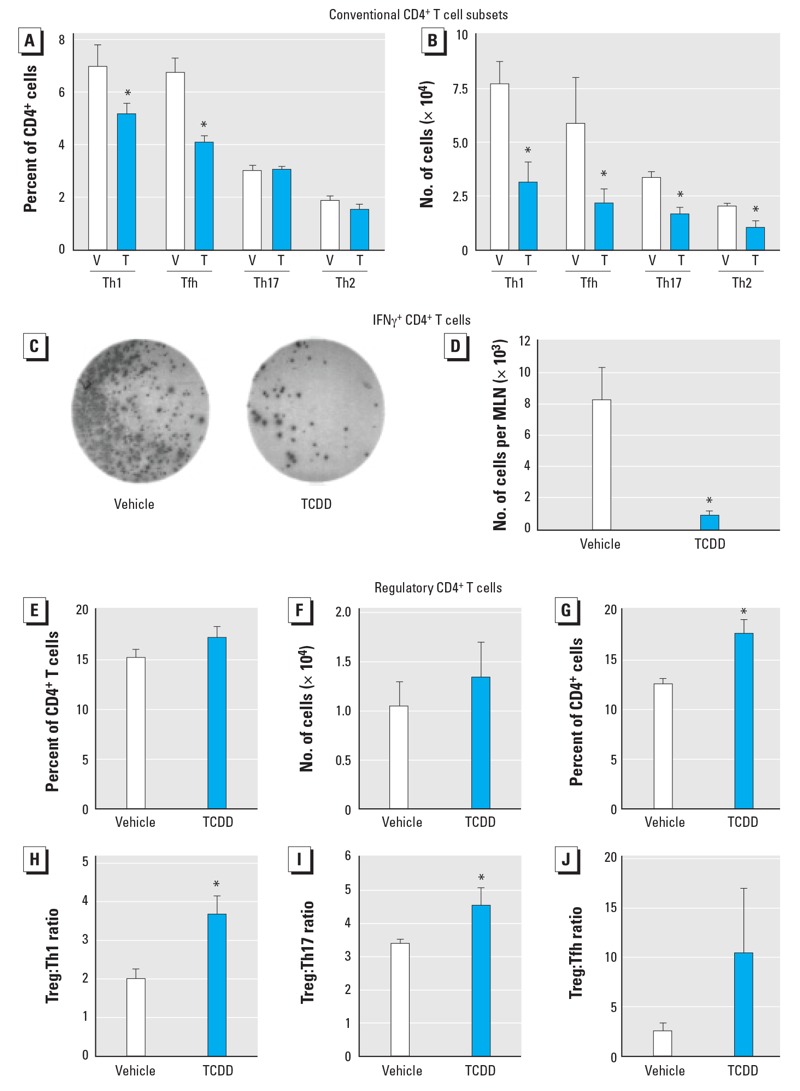
AhR activation during development altered CD4^+^ T‑cell subsets after IAV infection. Mice were developmentally exposed to vehicle (V) or TCDD (T) and infected with IAV as adults. The percentage (*A*) and number (*B*) of conventional CD4^+^ T‑cell subsets in the MLN enumerated by flow cytometry 9 days after infection: Th1 cells (Tbet^+^CD4^+^), Th2 cells (GATA3^+^CD4^+^), Th17 cells (RORγt^+^CD4^+^), and Tfh cells (CD44^hi^PD-1^+^CXCR5^+^CD4^+^) (see Supplemental Material, Figure S1). Representative wells (*C*) and number of IFNγ^+^CD4^+^ T cells/MLN (*D*) enumerated by the ELISPOT assay on day 9 postinfection. The percentage (*E*) and number (*F*) of Tregs prior to infection. The percentage of Tregs (*G*), and the ratio of Treg:Th1 (*H*), Treg:Th17 (*I*), and Treg:Tfh cells (*J*) in the MLN 9 days after infection. Values shown are mean ± SE; five or six female offspring from separate dams were used for each group. *p ≤ 0.05.

Upstream of T-cell–dependent antibody production, infection initiated CD4^+^ T-cell differentiation into several subpopulations of conventional effector cell subsets. Compared with infected offspring of control dams, infected offspring of TCDD-treated dams had a reduction in the frequency of all four conventional CD4^+^ T-cell subsets. Specifically, we observed a statistically significant decrease in the percentage of Th1 and Tfh cells (Figure 3A; see also Supplemental Material, Figure S1A,B). In addition, the number of Th1, Tfh, Th17, and Th2 cells in the MLN from infected mice developmentally exposed to TCDD was reduced by approximately 50% (Figure 3B; see also Supplemental Material, Figure S1A–D). We further examined the functional capacity of Th1 cells by determining the frequency of IFNγ^+^CD4^+^ T cells. The number of IFNγ^+^CD4^+^ T cells (Figure 3C,D) was significantly reduced in offspring developmentally exposed to TCDD, indicating that there were fewer Th1 cells, as defined using both phenotypic and functional makers.

Exposure of adult mice to AhR ligands has been reported to increase the frequency of Tregs (Quintana and Sherr 2013); hence, we examined whether triggering the AhR during development would have a similar effect. In the absence of infection, no discernable difference was observed in the percentage or number of Tregs in adult offspring of vehicle and TCDD-treated dams (Figure 3E,F; see also Supplemental Material, Figure S1E). However, after IAV infection the percentage of Tregs was increased in developmentally exposed offspring (Figure 3G). Unlike conventional CD4^+^ T-cell subsets, the number of Tregs was not decreased in the offspring of TCDD-treated dams. When all CD4 subsets were quantified from the same experiment, mice developmentally exposed to TCDD showed a decrease in conventional CD4^+^ T cells but an increase in Tregs. Therefore, the ratio of Treg:Th1, Treg:Th17, and Treg:Tfh cells was increased by developmental activation of the AhR (Figure 3H–J). Thus, in IAV-infected offspring of TCDD-treated dams, a greater proportion of the total CD4^+^ T-cell population was composed of Tregs than conventional effector CD4^+^ T cells.

**Figure 4:**
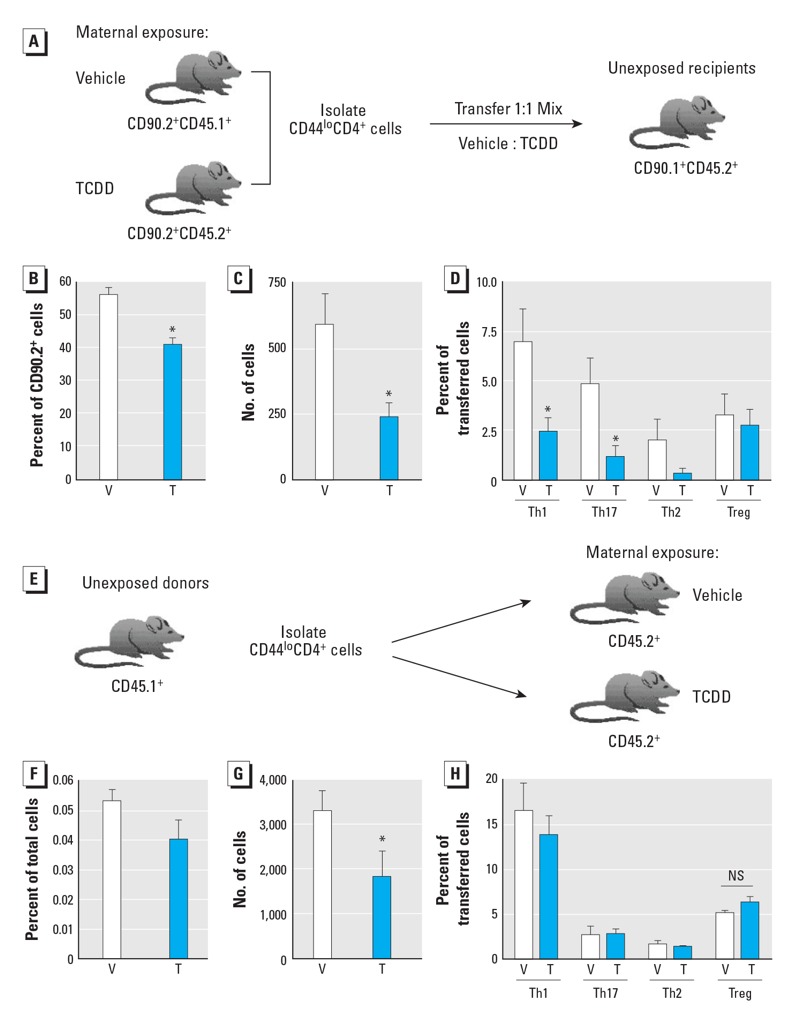
AhR activation during development leads to intrinsic changes in CD4^+^ T-cell responsiveness. (*A*) Dual adoptive transfer: Naive (CD44^lo^) CD4^+^ T cells (5 × 10^5^) from offspring of dams treated with vehicle (V) or TCDD (T) were transferred (1:1 ratio; 2.5 × 10^5^ cells/treatment) into unexposed congenic recipients; recipients were infected with IAV. The percentage (*B*) and number (*C*) of transferred CD90.2^+^ CD4^+^ T cells from each donor on day 9 postinfection. (*D*) The percentage of CD4^+^ T-cell subsets derived from each donor. (*E*) Single adoptive transfer: Naive CD4^+^ T cells (5 × 10^5^) from unexposed donors were transferred into congenic adult recipients that were developmentally exposed to vehicle (V) or TCDD (T); recipients were infected with IAV. The percentage (*F*) and number (*G*) of transferred CD4^+^ T cells, and (*H*) the percentage of effector CD4^+^ T-cell subsets derived from each donor on day 9 postinfection. Values shown are mean ± SE; 5–10 recipients were used per group per experiment. NS, no significant difference. *p ≤ 0.05.

To determine whether AhR activation during development changes CD4^+^ T cells in an intrinsic or extrinsic fashion, we performed reciprocal adoptive transfer experiments. In the dual adoptive transfer experiments, naive (CD44^lo^) CD4^+^ T cells from offspring developmentally exposed to TCDD (CD90.2^+^CD45.2^+^) or vehicle (CD90.2^+^CD45.1^+^) were combined in a 1:1 ratio and transferred into unexposed congenic recipients (CD90.1^+^CD45.2^+^) to ascertain whether triggering AhR during development causes intrinsic changes in CD4^+^ T cells (Figure 4A). The congenic markers allowed the two sets of donor-derived cells to be distinguished from each other and from recipient cells (see Supplemental Material, Figure S2A). The distribution of transferred CD4^+^ T cells from the two donor pools was not different (see Supplemental Material, Figure S2B). In separate experiments, we observed that developmental AhR activation caused the same alterations in CD4^+^ T-cell responsiveness to infection in all three congenic strains of mice (data not shown). On day 9 after IAV infection (the height of the CD4^+^ T-cell response), we observed a significant reduction in the percentage and number of CD4^+^ T cells in the MLN derived from donors developmentally exposed to TCDD (Figure 4B,C). In addition, of the transferred CD4^+^ T cells from donors developmentally exposed to TCDD, the percentage of cells that differentiated into Th1, Th17, or Th2 cells was reduced by approximately 2-fold, but there was no change in the percentage of Tregs (Figure 4D).

To examine potential extrinsic influences, we transferred naive CD4^+^ T cells from unexposed CD45.1^+^ mice into congenic (CD45.2^+^) adult offspring of dams treated with vehicle or TCDD (single adoptive transfer; Figure 4E). There were no differences in the transfer success rate or the distribution of transferred cells into naive recipients regardless of dam treatment (data not shown). Nine days after infection, there were fewer transferred CD4^+^ T cells in recipients that were developmentally exposed to TCDD compared with recipient offspring of vehicle-treated dams (Figure 4F,G). However, the percentage of conventional or regulatory effector CD4^+^ T-cell subsets was the same regardless of the developmental exposure of the recipients (Figure 4H). This is in direct contrast to observations in experiments in which the donor cells from developmentally exposed mice were transferred to unexposed recipients (Figure 4D).

## Discussion

The developmental basis of adult disease suggests that early-life exposures alter health and contribute to disease later in life. The mechanism by which developmental exposures lead to persistent changes in immune function is unknown, yet modifications to immunologically based processes have been reported to occur (Winans et al. 2011). Circumstantial evidence suggests that maternal exposure to AhR-binding chemicals impacts CD4^+^ T-cell–dependent processes and pathologies (Gehrs and Smialowicz 1999; Luster et al. 1980; Mustafa et al. 2011; Thomas and Hinsdill 1979; Vorderstrasse et al. 2006). We specifically examined CD4^+^ T cells, and observed that early-life AhR activation changed the proportion of functionally distinct CD4^+^ T-cell subsets responding to infection. These durable changes resulted from intrinsic and extrinsic effects on CD4^+^ T cells, indicating that AhR-mediated events in multiple cell types likely contributed to the immunomodulatory effects of developmental exposure. Specifically, changes in the ability of CD4^+^ T cells to differentiate into conventional effector subsets were due to effects that are intrinsic to CD4^+^ T cells. In contrast, an increase in the proportion of Tregs was likely due to a combination of intrinsic and extrinsic influences because the skewed frequency of Tregs was lost in both adoptive transfer scenarios. The requirement of both intrinsic and extrinsic consequences of developmental AhR activation on the increase in Tregs suggests that changes are required in both the CD4^+^ T cells and other cells of the organism. The overall expansion in CD4^+^ T-cell number after infection is likely also influenced by intrinsic and extrinsic factors because this effect was retained in reciprocal transfers. These data suggest that although AhR activation during development influenced extrinsic signals that can diminish the total number of CD4^+^ T cells responding to infection, these CD4^+^ T-cell–extrinsic events did not change the ability of the CD4^+^ T cells to differentiate.

A potential CD4^+^ T-cell lineage intrinsic effect of developmental AhR activation is a modification in epigenetic regulatory machinery. Major epigenetic programming events occur during development, and variations in the epigenetic marks laid down can impinge on cellular function (Cantone and Fisher 2013). Although epigenetic regulation in hematopoietic cells remains poorly understood, epigenetic mechanisms influence CD4^+^ T-cell development and function. For example, CD4^+^ T-cell function has been reported to be altered by changes in DNA methylation, histone modifications, and other manipulators of chromatin structure (Brand et al. 2012; Carson et al. 2010). Also, a few studies have reported that AhR activation altered the pattern of epigenetic marks in other model systems (Manikkam et al. 2012; Papoutsis et al. 2013; Singh et al. 2011). Thus, it is plausible that AhR activation during development can change epigenetic regulatory machinery in CD4^+^ T cells, leading to intrinsic differences when these cells respond to viral infection.

Potential extrinsic factors include other immune cells, such as antigen-presenting cells (APCs) and B cells, which interact bidirectionally with CD4^+^ T cells to shape the response to infection (Alam et al. 2014; Smith et al. 2004). B cells and APCs express the AhR, and their function is modulated by AhR activation in adult animals (Jin et al. 2014; Quintana and Sherr 2013; Sulentic and Kaminski 2011). Non-hematopoietic cells are also important for CD4^+^ T-cell development and function (Mebius 2003). Although not examined in the context of developmental exposure, AhR ligands have been reported to modulate immune function via direct effects on non-hematopoietic cells (Camacho et al. 2005; Jensen et al. 2003; Wheeler et al. 2013). Also, in the context of developmental exposure, TCDD altered pulmonary inflammation after infection via effects extrinsic to immune cells (Hogaboam et al. 2008). Therefore, AhR-mediated changes in other leukocytes and in non-hematopoietic cells may contribute to alterations in CD4^+^ T-cell responses in developmentally exposed offspring.

It has long been known that developmental exposure to AhR ligands suppresses antibody responses in the offspring. However, much of the prior data was generated using higher maternal or perinatal doses that caused transient thymic atrophy or other signs of hematotoxicity (Faith and Moore 1977; Thomas and Hinsdill 1979; Vos and Moore 1974). Other studies used maternal doses of TCDD only slightly higher than those used in the present study, and those doses decreased CD4^+^ T-cell–dependent and independent antibody responses (Vorderstrasse et al. 2006). In the present study, we used a lower, more environmentally relevant dose of TCDD, which reduced CD4^+^ T-cell–dependent, but not independent, antibody responses to IAV. Also, we observed that the frequency of Tfh and Th1 cells, key contributors to T-cell–dependent antibody and antiviral responses, were correspondingly diminished. This suggests that CD4^+^ T-cell functions may be more sensitive to perturbation by developmental exposure to AhR ligands than are functions of other immune cell types, such as B cells. Thus, this work has implications for examinng how early-life exposures affect immune function in the human population, where antibody responses are often the sole measurement. Although it is challenging to equate doses in animal models to human exposures, the maternal dose we administered did not cause overt toxicity, which is consistent with reports that exposed human populations present changes in immune function without obvious changes in immune organ cellularity. For instance, epidemiological data indicate that early-life exposures to AhR ligands are correlated with decreases in vaccine-specific IgG levels in children, which require a robust CD4^+^ T-cell response (Heilmann et al. 2010; Hochstenbach et al. 2012; Stølevik et al. 2013). Therefore, it may be important to isolate and more closely examine CD4^+^ T-cell responses in future studies with human cohorts.

In the present study, we focused on the response of CD4^+^ T cells to primary acute infection with a common human pathogen. Yet, the implications extend beyond IAV. For example, *Toxoplasma gondii*, *Streptococcus pneumoniae*, and *Mycobacterium tuberculosis* require CD4^+^ T cells for pathogen clearance (Cohen et al. 2013; Leveton et al. 1989; Malley et al. 2005). In addition, pathogens that have not been major burdens because of successful immunization strategies may reemerge if vaccine efficacy is reduced by exposures experienced during development. Furthermore, many autoimmune and allergic diseases are CD4^+^ T-cell dependent, such as asthma (Vock et. al. 2010) and multiple sclerosis (Dittel 2008), suggesting that these diseases may be altered by developmental exposures to AhR ligands. This idea is supported by studies showing that developmental exposure to TCDD enhanced autoimmune symptoms later in life (Mustafa et al. 2011). Therefore, in addition to indicating that developmental activation of the AhR directly impinged upon the function of CD4^+^ T cells in the context of infection, results of the present study suggest that AhR-mediated events in CD4^+^ T cells may be an important underlying factor in other infectious and immune-mediated diseases.

## Conclusions

We observed that developmental exposure to AhR ligands caused lasting changes in CD4^+^ T-cell responses to infection due to direct effects on the CD4^+^ T-cell lineage. These results have global implications because CD4^+^ T cells are critical in appropriate immune responses to many pathogens and vaccines.

## Supplemental Material

(334 KB) PDF
