# Supplementary material for: Effects of Developmental Activation of the AhR on CD4+ T-Cell Responses to Influenza Virus Infection in Adult Mice
Source: Environ Health Perspect. 2014 Jul 22;122(11):1201–8. doi: 10.1289/ehp.1408110 (PMC4216167; doi:10.1289/ehp.1408110)
Supplement: (334 KB) PDF [file ehp.1408110.s001.508.pdf]

Environmental Health Perspectives doi:10.1289/ehp.1308110

Supplemental Material

Effects of Developmental Activation of the AhR on $CD4^+$ T-Cell Responses to Influenza Virus Infection in Adult Mice

Lisbeth A. Boule, Bethany Winans, and B. Paige Lawrence

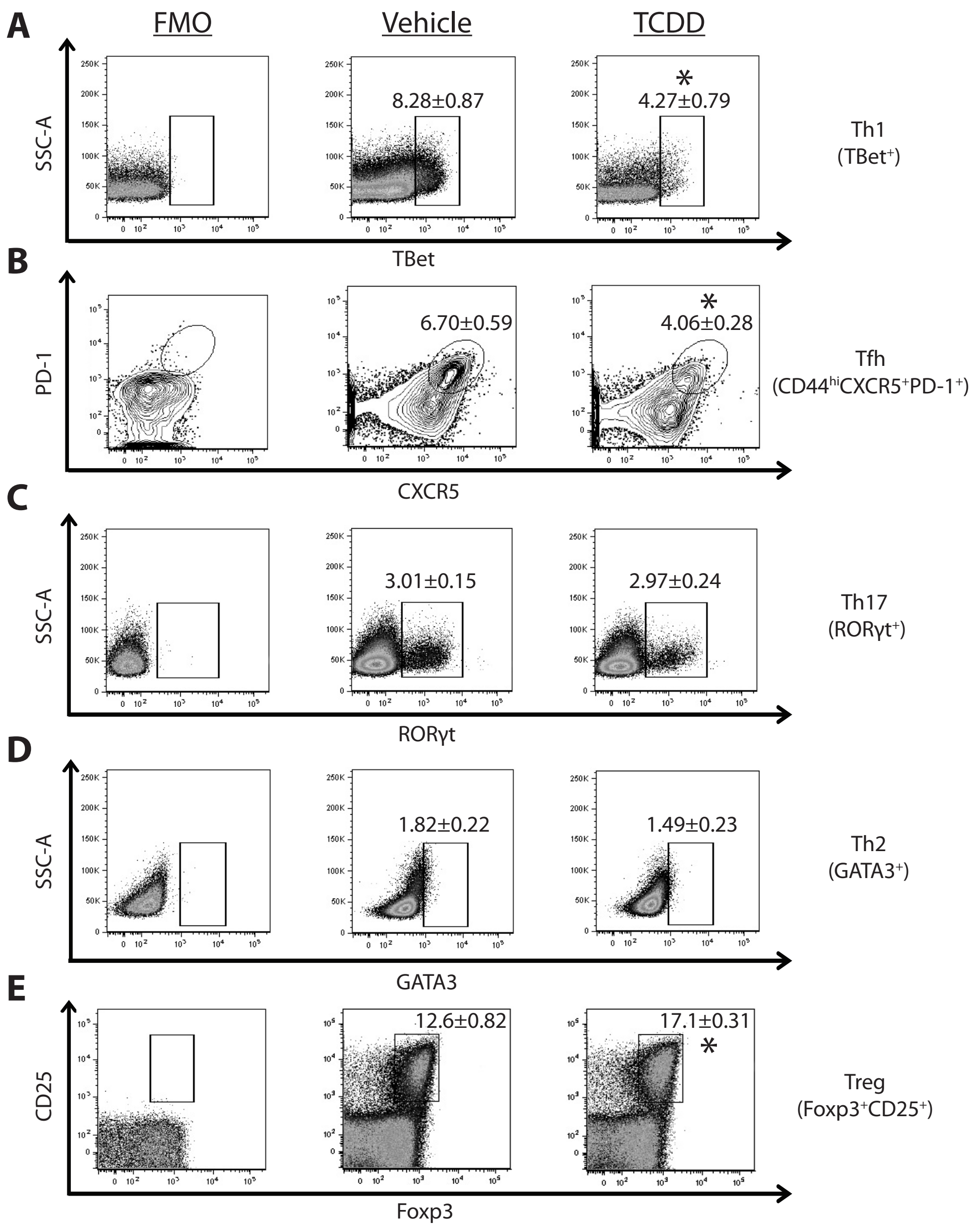

Figure S1. Activation of the AhR during development leads to changes in the percentage of multiple $CD4^+$ T cell subsets after influenza virus infection. Developmentally exposed mice were infected (i.n.) with IAV and MLNs were harvested on day 9 post infection. Cells were co-stained with antibodies for multi-parametric flow cytometry. The dot plots show the gating strategy for each $CD4^+$ T cell subset. Prior to gated regions depicted, doublets and non-viable cells were excluded, and $CD4^+$ T cells were defined as cells that were $CD3^+CD4^+$. As such, all dot plots

depicted are gated on $CD4^+$ T cells, except the Tfh plots, which are further gated on $CD44^{hi}CD4^+$ T cells. The dot plots depict the percentage following populations: (A) Th1 cells, (B) Tfh cells (C) Th17 cells, (D) Th2 cells and (E) Treg cells. The percentage of each population (out of total $CD4^+$ T cells) is displayed on each plot (± SEM). The left column shows dot plots of the fluorescence minus one (FMO) controls for each staining set. The middle column contains representative dot plots of data from a mouse developmentally exposed to vehicle, and the right column shows representative dot plots of data from a mouse developmentally exposed to TCDD. The number of cells in each sub-population was calculated by multiplying the percentage by the total number of viable MLN cells or $CD4^+$ T cells (cell number determined using TC10 Automated Cell Counter, BioRad, Hercules, CA) Five to six sex matched mice from different dams were used at each time point. Experiments were independently repeated with separate dams and yielded similar results. An * signifies $p < 0.05$.

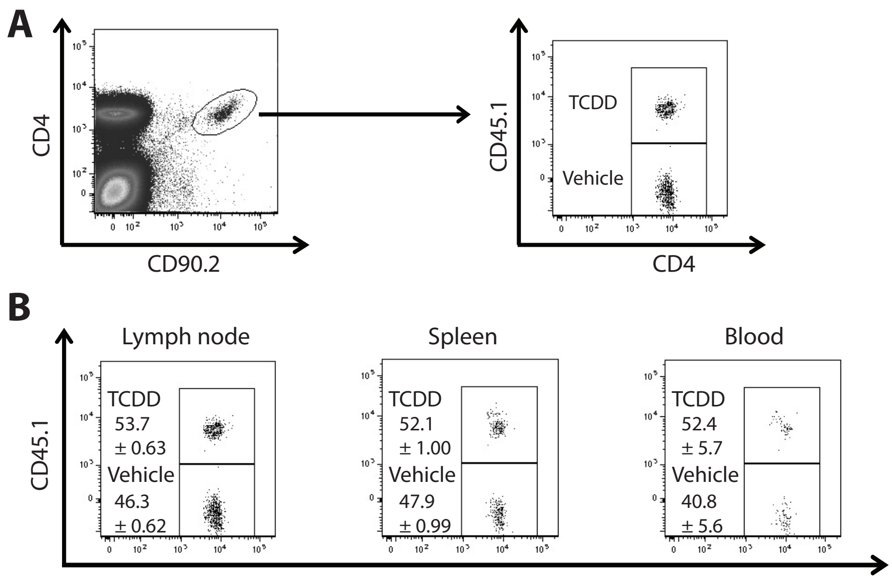

Figure S2. AhR activation during development does not change the in vivo distribution of transferred naïve $CD4^+$ T cells. (A) Gating strategy for dual adoptive transfer experiments. Transferred cells are defined as $CD4^+CD90.2^+$ cells. Those derived from mice developmentally exposed to vehicle are $CD4^+CD90.2^+CD45.1^-$ and those from donors developmentally exposed to TCDD are $CD4^+CD90.2^+CD45.1^+$. (B) Representative dot plots show the average percentage (± SEM) of $CD4^+$ T cells from mice developmentally exposed to vehicle ($CD45.1^-$; bottom) and TCDD ($CD45.1^+$; top) in the lymph nodes, spleen, and blood of naïve recipients (36 hr post transfer).
